# Cyanobacterial Harmful Algal Bloom Toxin Microcystin and Increased *Vibrio* Occurrence as Climate-Change-Induced Biological Co-Stressors: Exposure and Disease Outcomes via Their Interaction with Gut–Liver–Brain Axis

**DOI:** 10.3390/toxins15040289

**Published:** 2023-04-17

**Authors:** Saurabh Chatterjee, Madhura More

**Affiliations:** 1 Environmental Health and Disease Laboratory, Department of Environmental and Occupational Health, Program in Public Health, University of California–Irvine, Irvine, CA 92697, USA; 2 Toxicology Core, NIEHS Center for Oceans and Human Health on Climate Change Interactions, Department of Environmental and Occupational Health, Program in Public Health, University of California–Irvine, Irvine, CA 92697, USA; 3 Division of Infectious Disease, Department of Medicine, UCI School of Medicine, University of California–Irvine, Irvine, CA 92697, USA

**Keywords:** harmful algal blooms, microcystin, resistome, microbiome, gut–liver–brain axis, metabolic disease

## Abstract

The effects of global warming are not limited to rising global temperatures and have set in motion a complex chain of events contributing to climate change. A consequence of global warming and the resultant climate change is the rise in cyanobacterial harmful algal blooms (cyano-HABs) across the world, which pose a threat to public health, aquatic biodiversity, and the livelihood of communities that depend on these water systems, such as farmers and fishers. An increase in cyano-HABs and their intensity is associated with an increase in the leakage of cyanotoxins. Microcystins (MCs) are hepatotoxins produced by some cyanobacterial species, and their organ toxicology has been extensively studied. Recent mouse studies suggest that MCs can induce gut resistome changes. Opportunistic pathogens such as *Vibrios* are abundantly found in the same habitat as phytoplankton, such as cyanobacteria. Further, MCs can complicate human disorders such as heat stress, cardiovascular diseases, type II diabetes, and non-alcoholic fatty liver disease. Firstly, this review describes how climate change mediates the rise in cyanobacterial harmful algal blooms in freshwater, causing increased levels of MCs. In the later sections, we aim to untangle the ways in which MCs can impact various public health concerns, either solely or in combination with other factors resulting from climate change. In conclusion, this review helps researchers understand the multiple challenges brought forth by a changing climate and the complex relationships between microcystin, *Vibrios*, and various environmental factors and their effect on human health and disease.

## 1. Cyanobacteria and Cyano-HABs

Cyanobacteria are unicellular, prokaryotic, photoautotrophic, Gram-negative bacteria found in fresh and brackish waters. Some commonly found genera of cyanobacteria are *Microcystis*, *Nostoc*, *Dolichospermum*, and *Planktothrix* [1]. Cyanobacteria are the oldest known micro-organisms that photosynthesize and release oxygen. Their contribution to the current state of the Earth’s environment is immense [2]. Under favorable nutrient, temperature, and water flow conditions, cyanobacteria and other phytoplankton grow excessively and cause bloom formation. When these blooms have a harmful effect on the water ecosystem and the surrounding environment by means of toxin production, they are considered harmful algal blooms (HABs) [3]. Around the world, various cyanobacterial harmful algal bloom (cyano-HAB) events have led to massive animal and human health problems, ecological disturbances, and economic losses [4,5,6]. Cyano-HABs make drinking water unsuitable for consumption, cause habitat loss for other aquatic species, cause health problems in the animals and humans that are exposed to them, inflict economic losses due to the shutdown of aquaculture enterprises, and generally reduce the aesthetic quality of lakes and water bodies that are tourist attractions [1,7,8,9]. Some cyanobacterial species produce a variety of toxins, including hepatotoxins such as microcystins (MCs) and cylindrospermopsin, neurotoxins such as saxitoxins and anatoxins, and endotoxins such as lipopolysaccharide and lyngbyatoxins [10,11,12,13]. These toxins pose a major threat to the use of these water bodies as sources of drinking water, irrigation, and for recreational purposes. The ingestion of these toxins causes human and animal health problems such as liver and gastrointestinal disorders, skin diseases, and neurological impairment [10,11,13]. 

After cyano-HABs subside, the amount of organic matter left in the water body is enormous, increasing their biological and chemical oxygen demand. Eventually, the water body turns into a dead zone incapable of supporting the growth of aquatic flora and fauna [14,15]. They also release chemicals such as geosmin, which are responsible for musty odors in the vicinity [16].

## 2. Climate Change and Cyanobacterial HABs

Predictions about the changing climate, which is changing due to global warming, can potentially promote the rise in frequency, geographical expanse, and duration of HABs and cyano-HABs in bodies of water [17,18]. Conditions associated with climate change, including an increase in surface temperatures, increased atmospheric carbon dioxide, eutrophication, the increased salination of freshwater due to frequent droughts, rising sea levels, and changes in local climate patterns, contribute to the development of favorable conditions for cyano-HABs [18]. O’Riley et al. conducted an extensive analysis using a database consisting of the surface water temperatures of lakes in the summer and climate variables derived from in situ and/or satellite measurements from 235 lakes worldwide. They found that globally, the summer surface temperatures of the lakes rose by 0.34 °C from 1985 to 2009 [19]. Further, future projections of daily water temperature, produced using global hydrological water temperature modelling and bias-corrected for the intergovernmental Panel on Climate Change (IPCC) Special Report on Emissions Scenarios (SRES) A2 and B1, indicate that for both scenarios, the global mean and highest water temperatures are projected to increase by 0.8–1.6 °C in 2071–2100 relative to 1971–2000 [20]. Cyanobacteria exhibit optimal growth at elevated temperatures (>25 °C). At these temperatures, cyanobacteria effectively outcompete other eukaryotic phytoplankton [21].

An increase in the surface water temperature of fresh and marine water systems leads to a reduced density of the surface water, thus preventing the vertical movement of the surface water. Thus, long and intense periods of warm temperature strengthen and increase the duration of the vertical stratification of marine and freshwater systems [22,23]. Cyanobacterial strains can use this vertical stratification to their advantage by regulating their buoyancy through the formation of intracellular gas vesicles [24,25]. Increased irradiation of the surface water is efficiently absorbed by cyanobacteria with minimal photosynthetic damage due to the composition of photoprotective accessory pigments [26,27,28] and light-induced changes in photosystem II (PSII) [29]. Further, during a bloom, cyanobacteria can prevent sunlight penetration to deeper layers of the water system, which can prevent the normal growth of eukaryotic phytoplankton.[30].

**Figure 1:**
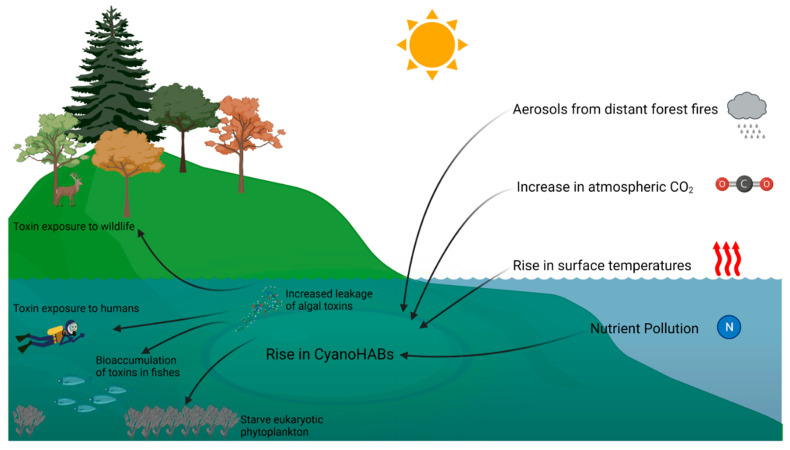
Effects of climate change on cyanobacterial harmful algal blooms and their consequences.

Increased carbon dioxide in the atmosphere is advantageous to cyanobacterial photosynthesis and growth [31,32]. An increase in the salt concentrations of lakes, reservoirs, riverine areas, and estuaries due to a rise in sea levels and frequent droughts further leads to vertical density stratification, which is advantageous for buoyant cyanobacteria (Figure 1) [18]. Further, many species of freshwater cyanobacteria such as *Anabaena*, *Microcystis*, and *Nodularia* are halotolerant. A temporary increase in salt concentration of up to 7.5 g/L is tolerable by toxin-producing cyanobacteria but leads to an increased leakage of toxins into the surrounding environment to prevent osmotic damage [33,34]. This leads to localized high concentrations of toxins in the cyano-HABs. This phenomenon is often observed in cyano-HABs in brackish waters, as the salt concentration there is higher due to the proximity to sea water [35].

Periods of intense rainfall followed by periods of severe drought and increases in nutrient runoff from agricultural land and industries into bodies of water cause the eutrophication of the bodies of water over a longer period [36]. A comparison of National Lake Assessments Reports from 2007 to 2017 and other similar assessments indicate that nutrient pollution in the lakes in the USA is worsening. Of these lakes, 45% demonstrated elevated nitrogen levels, and 46% of the lakes demonstrated elevated phosphorous levels [37,38,39]. Eutrophication plays a key role in the rise of cyano-HABs.

Thus, data from the fallout of climate change related to severe drought, increased levels of nutrients in runoff, and eutrophication predict a future environment conducive to an increased frequency and intensity of cyano-HAB blooms [15].

## 3. Increase in Cyanotoxin Exposure: Microcystin

Studies using batch, semi-continuous, and continuous algal culture systems show that an increase in surface temperatures [40,41]), an increase in light intensity (up to 60 μmol photons m^−2^ s^−1^) [42,43,44,45], and an increase in the cyanobacterial growth rate [46,47], aided by excess nitrogen [48,49,50,51,52], have the potential to increase the rate of MC-LR production. However, the effect of an increase in CO_2_ levels on toxin production is not clear. Under the condition of a simultaneous excess of nitrogen and inorganic carbon in algal culture, there was an increase in the production of MC, particularly MC-RR [52], while other studies note that an increase in the levels of atmospheric CO_2_ favors the growth of non-toxin-producing strains under conditions of low light [53]. Therefore, rising cyano-HABs could also potentially lead to an increased concentration of cyanotoxins such as MCs in bodies of water and consequently in drinking water. Microcystin was detected in 21% of the lakes in the USA. Microcystin levels rose above the recreational water quality criterion recommended by the Environmental Protection Agency (EPA) in 2% of lakes across the nation, roughly 4400 lakes [37].

Microcystins exist in various congeners [54]. There are several reports that indicate the presence of MCs and MC-LR in drinking water reservoirs and water treatment plants from different parts of the world [55,56,57]. A study was conducted at two drinking water treatment plants in Brazil with contrasting water treatment technologies for the removal of MCs. The risk and expected number of days for which infants, children, and adults would be exposed to dissolved MCs concentrated in drinking water at above the thresholds established by the World Health Organization (WHO) and the United States Environmental Pollution Agency (USEPA) were computed in the form of hazard quotients (HQs). The HQs indicated that through the drinking water, individuals of all ages at one of the two water treatment facilities were at a higher risk of exposure to dissolved MCs at above the established threshold values [55]. This study indicates that infants who consume more water than adults or older children are particularly vulnerable to the toxic effects of MCs [56]. Similar studies conducted in drinking water treatment plants in Quebec (Canada) and Kubani (Nigeria) also noted HQs greater than 1, even after drinking water treatment [58]. Water samples from twenty-four water reservoirs in Eastern Cuba were evaluated for MC-LR levels. Concentrations exceeding the WHO limits for drinking water (1 µg/L) were detected in nine out of the twenty-four reservoirs. These lakes were also affected by eutrophication, higher surface water temperatures, and high phytoplankton cell concentrations, particularly cyanobacteria [57]. The presence of cyanobacteria and cyanotoxins was investigated in the Amazon River at a drinking water treatment plant. Both cyanobacteria and cyanotoxin levels were low throughout the year except in June–August in 2015, when the MC-LR concentration exceeded the established threshold values (2.1 µg L^−1^), coincident with a peak in the density of the cyanobacteria *Limnothrix planctonica* [59]. A study conducted in Qatar analyzed drinking water from seven water tanks in urban areas of Qatar and twelve water sources, such as desalination plants and groundwater wells, from rural areas in Qatar. In three samples from the urban areas, the MC-LR concentration exceeded the WHO guideline, ranging from 0.66 µg/L to 1.33 µg/L in the samples collected from urban areas. The MC-LR concentration ranged from 0.65 µg/L to 0.89 µg/L in the samples collected from rural areas [60]. In a study conducted in Morocco, extracted and lyophilized biomass from lakes with cyano-HABs was used to expose mice, and the toxicity in mice was determined by calculating the lethal dose 50 (LD50). It was found that the toxicity in mice was positively associated with the concentration of MC-LR in the biomass, cyanobacterial cell density, and the level of eutrophication in the lake [61]. Water bodies with a higher cyanobacterial cell density have been noted to have higher MC-LR concentrations (Table 1). Further, an increased temperature (>20 °C) favors the release of MCs [62].

A review of selected articles on cyanotoxin occurrence and animal or human poisonings that were published prior to 2018 was performed by Svirčev et al. Out of the 1118 records identified, 699 records reported MCs as one of the cyanotoxins. This accounts for 63% of all cyanotoxin reports. MCs were widely reported amongst all cyanotoxins, followed by cylindrospermopsin (10%), anatoxins (9%), and saxitoxins (8%). Microcystins are the most frequently reported cyanotoxins worldwide [65]. Svirčev et al. conducted a review of 134 published reports and epidemiological investigations from 1960 to 2016 of human health incidents associated with mass population exposures to cyanobacteria. However, because of the scattered nature of the report, definitive conclusions on the effect of MCs in the emergence of global health effects could not be made. The major health outcomes associated with cyanobacterial exposure were chronic and acute morbidity, cancers, mortality, and birth outcomes [66].

Across the world, epidemiological studies have linked microcystin exposure to various health outcomes. Most epidemiological studies on the public health consequences of MC exposure have been conducted in China. These studies positively link MC exposure with incidences of primary liver cancers (PLC) [67], hepatocellular carcinoma (HCC) [68], colorectal cancer (CRC) [69], and stomach cancer. Microcystins have been reported to affect multiple organ systems beyond their usual role as a hepatotoxin. The lungs, skin, and the gastrointestinal tract are affected, as shown in various animal models [10]. We have previously shown that MCs target protein phosphatase IIA in the fatty liver and can enhance inflammation via a NADPH-oxidase-derived mechanism that also involves triggering NLRP3 activation [70]. Studies conducted in Serbia from 1998 to 2008 found an association of MC exposure with PLC, hepatitis B and C [71], liver cirrhosis [71], and 10 kinds of cancers, including cancers of the brain, heart, ovary, testis, stomach, colon, lung, and skin [72]. Studies conducted around the globe have also positively linked exposure to MC-producing cyanobacteria such as *Microcystis aeruginosa*, *M. flos-aquae*, *Aphanizomenon flos-aquae*, *Anabaena flos-aquae*, *A. spiroides*, and *Planktothrix agardhii* with incidences of cancers of the liver, colon, and rectum and hepatitis B and C [66]. Further, MC-LR serum levels increased the risk of tumor relapse in HCC patients and were associated with higher mortality. This poor prognosis coincided with elevated oxidative stress in HCC patients exposed to MC [73]. Further, MC exposure alone and in combination with other known risk factors, such as cadmium, contributed to an elevated risk for chronic kidney disease, according to study conducted in central China. This study identified MC-LR as an independent risk factor for CKD [74]. In a cross-sectional study in southwest China, abnormal renal function indicators were linked with higher mean serum levels of MC-LR [75].

The contribution of MC-LR towards the progression of liver diseases, chronic kidney disease, and cancer progression is immense, as indicated by epidemiological data. Some of these associations have been mechanistically validated in animal models. Therefore, for this review, we choose to focus on MC as a representative of the impact of increasing HABs in underlying diseases such as vibriosis, nonalcoholic fatty liver disease, type II diabetes, and cardiovascular diseases and its contribution to other stressors that complicate disease prognosis, such as heat stress and antibiotic resistance.

In this review, we will investigate the effects of climate change on the increase in cyano-HABs and their role in promoting antibiotic resistance and complicating infections caused by opportunistic pathogens, such as *Vibrio vulnificus*, *Vibrio parahaemolyticus*, and other *Vibrio* species, as their increase in abundance also is closely linked to climate change and its effects [76,77,78,79,80].

*Vibrio* spp. is an estuarine marine bacterium ubiquitously present in all aquatic organisms and habitats [81]. Studies indicate a trend of an increase in the presence and rate of infection by *Vibrio* in the USA parallel to a trend in an increase in the atmospheric temperature [82]. Another factor that can affect the growth of *Vibrio* spp. is salinity. With rising sea levels, the salinity of fresh and brackish water systems is increasing [82]. This aids the expansion of *Vibrio* spp. into previously unreported aquatic systems such as coastal floodwaters, increasing the chances of human exposure via recreational activities and through drinking water. Therefore, climate change due to global warming is projected to greatly facilitate the growth of *Vibrio* spp. temporally and spatially [83]. When considering the rise in harmful cyano-HAB-producing toxins and the increase in the abundance and infection rates of *Vibrio* spp. with the predictions for climate change, Vibrio–MC co-exposures may not be a rare event, although the routes of exposure for MC and *Vibrio vulnificus* and *Vibrio parahaemolyticus* are not identical. The major MC exposure route is through drinking water, and the major exposure route for *Vibrios* lies in contaminated seafood or dermal contact with contaminated waters. Co-exposures via distinct routes may increase in the future, as an increase in toxin-producing and non-producing cyanobacterial algal mass may support the growth of *Vibrios* in estuarine, brackish, and coastal water detention systems [76,77,78,79,80]. A study that used data from coastal detention pond systems in South Carolina, USA, found that the HABs in these ponds were associated with increased abundances of *V. vulnificus* and *V. parahaemolyticus* only when the water temperatures were warmer (>10 °C) [84]. The abundance of these bacteria increased following blooms caused by cyanobacteria and dinoflagellates. *Vibrio* spp. are opportunistic and can use substrates such as phytoplankton as attachment sites when they are abundant in bloom conditions [84]. Extreme weather conditions such as hurricanes can further increase the abundance of these pathogens in coastal waters [78]. Further, fishers and pisciculture workers are at an elevated risk of these co-exposures, as cyanobacteria [85,86] and *Vibrios* [87,88,89] are abundantly found in these water samples despite the absence of bloom conditions [90,91].

We further explore manifestations of MC toxicity in underlying conditions such as heat stress, cardiovascular diseases, type II diabetes, and non-alcoholic fatty liver disease. In the following sections of this review, we use MC as an example to demonstrate the profound consequences of the rise in cyano-HABs in humans and animals. Further, we aim to discuss the plausible connections between two climate-change-related challenges, namely, MC exposure and exposure to pathogenic *Vibrio* spp. This review is unique as it considers together the effect of multiple climate-change-induced changes, such as an increase in cyanobacterial blooms, an increase in heat stress, an increase in vibrio infections, and the concurrent increase in various lifestyle-associated diseases such as cardiovascular disease, non-alcoholic fatty liver disease, and type II diabetes. Thus, the review identifies crucial areas for future research to assess the impact of the changing climate and a combination of HABs, Vibrios, and periodic heat waves on human health.

## 4. General Toxicity of Microcystins

**Figure 2:**
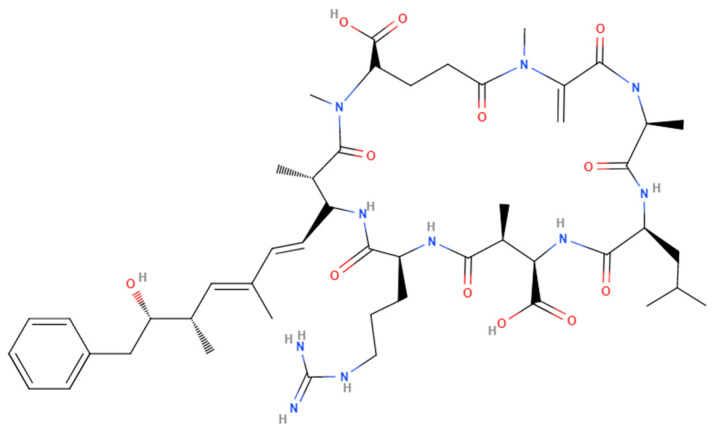
Chemical structure of Microcystin-LR.

MCs are ubiquitous toxic secondary metabolites produced by photoautotrophic Cyanobacteria [65]. The microcystin ABC (*mcyABC*) cluster is responsible for the biogenesis of MCs in various aquatic cyanobacteria belonging to the genera *Microcystis*, *Anabaena*, *Plankothrix*, *Hepalosiphon*, and *Nostoc*. Chemically, MCs are monocyclic heptapeptides and require organic anion transporting polypeptides (OATPs) such as OATP1B1, OATP1B3, and OATP1A2 for their uptake. More than 240 MC congeners exist, depending on different amino acids at positions 2 and 4 of the heptapeptide and the methylation status of D-methyl aspartic acid (D-MeAsp) and N-methyl dehydroalanine (Mdha). The polarity of the MC molecule is determined by the variable amino acids at positions 2 and 4 of the heptapeptide (Figure 2). The 3-amino-9-methoxy-2,6,8-trimethyl-10-phenyldeca-4,6-dienoic acid (ADDA) subunit is responsible for the inhibition of serine/threonine protein phosphatases, which is the key mechanism of MC toxicity, depending on the identity of the variable amino acids at positions 2 and 4. Some common MC congeners include MC-LR, MC-RR, MC-YR, and MC-LA [92].

Human exposure to MCs commonly occurs through the consumption of drinking water, contaminated fish, shellfish, vegetables, and algal dietary supplements and through recreational activities near bodies of water affected by MC-producing cyanobacterial HABs [93,94,95]. MC-LR is the most abundant and extensively studied MC. It is the only MC congener to have a daily intake limit set at 0.04 µg/ kg bodyweight for adults, as established by the World Health Organization (WHO). For an adult weighing 60 kg, the safe estimated daily intake (EDI) for MC-LR is 2.4 µg/kg [96]. MC exposure through food also warrants attention. In fruits and vegetables irrigated with contaminated waters, the total MC concentration was found to be approximately 382 μg/kg fresh weight. Most vegetable samples surveyed in this study posed a risk to humans of exceeding the EDI limit if accounted for with the reference dose for oral exposure of MC-LR (0.04 μg/kg/day) [97]. Fish consumption can be a dominant route of human exposure to MCs. In a study conducted in freshwater lakes, which included some lakes supporting fisheries in Uganda and North America, it was found that MC bioaccumulation in fish increased with an increase in algal biomass. The potential daily exposure to MC for a 60 kg daily consumer of water and fish from a majority of the lakes under study would exceed the WHO estimated daily safe intake limit of (2.4 μg/day) [98]. Therefore, MC exposure occurs at multiple trophic levels due to its bioaccumulation in fish, seafood, fruits, and vegetables.

The liver is the primary site of MC distribution [99]. MCs are stable and accumulate in the liver in all animal models and in humans. Robinson et al. demonstrated that the percentage of an MC dose in the liver after intravenous administration in rats did not change from 2 h up to 7 days post exposure [100]. This effect can be attributed to the efficiency of rat hepatic OATPs or a lack of robust efflux mechanisms. Species-specific differences exist in MC absorption and distribution that contribute to their differential toxicities. The LD_50_ of MC-LR in mammals is 100 times lower than in fishes [101]. Human albumin has the highest affinity for MC-LR binding compared to bovine, porcine, and fish albumin. Hence, MC toxicokinetics in humans may differ from animal models [101]. A transgenic mouse model expressing human variants of OATPs and efflux proteins can be used to better understand MC toxicokinetics in humans.

MCs have strong hepatotoxic and genotoxic properties [102]. Additionally, studies conducted in animal models and in cell lines demonstrated their neurological [103], renal [104], and reproductive toxicities [105]. MCs primarily inhibit serine/threonine protein phosphatases PP1 and PP2A by binding to the catalytic subunit of the proteins [106]. The abnormal phosphorylation of the downstream protein substrates of PP1 and PP2A leads to cellular apoptosis, cytoskeletal destabilization, increased inflammation, the disruption of DNA damage repair, and tumorigenesis [107]. Additionally, MC also directly interacts with mitochondrial proteins, triggering oxidative stress and apoptosis [108]. Therefore, MC toxicity involves a network of different signaling pathways [109,110].

**Figure 3:**
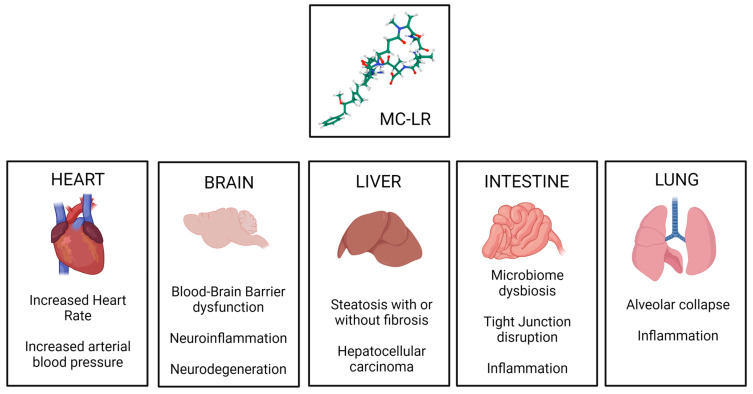
Organ-specific toxic mechanisms of Microcystin, established using various animal models.

Previous studies have shown that MC exposure leads to gut microbiome dysbiosis, causing disruption in intestinal permeability [111]. MC further exacerbates fatty liver disease and aids in the progression of the disease to aggressive forms, such as steatohepatitis and liver cirrhosis [112]. MC exposure also leads to extra hepatic complications such as neuronal inflammation, blood–brain barrier dysfunction [113], and renal toxicity (Figure 3) [114]. Recent data from mouse studies indicate that gut dysbiosis due to MC exposure can cause changes in the gut resistome, increasing the abundance of antibiotic-resistant genes in the gut bacteriome. These changes have been linked to the release of proinflammatory cytokines and increased signs of immunosenescence (Table 2) [111].

## 5. Exposure to Microcystins Has a Potential to Complicate Pathology in Underlying Diseases and Parallel Conditions That Are Affected by Climate Change

Is increasingly being recognized that climate change affects many facets of health. Potential areas in which the effects of climate change on human health may result in exacerbated conditions include heat-related mortality, food insecurity, reduced crop yields, increased suitability for the transmission of infectious diseases, rising sea levels, cardiovascular morbidity, mortality from increasingly severe wildfires, and the myriad health effects resulting from other extreme weather events such as floods, coastal floods carrying a risk of exposure to vibrio blooms to fresh and brackish water, and droughts [123]. The Gut-Liver Axis plays a crucial role in mediating the toxic effects of MC-LR. MC-LR causes gut dysbiosis with or without the presence of underlying liver disease [111]. Gut dysbiosis due to MC-LR with underlying liver diseases further exacerbates inflammation in the liver [114], brain [113], and kidney [114,124]. Further, early life exposure to MC-LR in mice cause gut dysbiosis and predisposes them to metabolic disorders like type 2 diabetes, non-alcoholic fatty liver disease and obesity [125].

### 5.1. Antibiotic Resistance

Antibiotic resistance has been viewed as a significant event that is affected by climate change or bears a strong association with the changing climate [123]. Antibiotic resistance (AR) is one of five factors that severely affect the world economy [126]. It is one of the greatest challenges faced by modern medicine and is a major public health crisis. Hence, studying the sources of AR and environmental factors that affect the abundance and transmissibility of AR genes is important [126,127]. Any environmental niche that harbors large numbers of bacteria is a potential source for AR gene transfers. The gut harbors trillions of bacteria and is an ideal environment for the development of mechanisms of antimicrobial resistance and their transmission. The collection of all genes that contribute to antibiotic resistance from all bacteria in the human microbiome is called the human antibiotic resistome [128]. The resistome includes AR genes and cryptic resistance genes which are not always expressed in pathogenic and non-pathogenic bacteria, including antibiotic producers [129]. The resistome also includes those genes encoding proteins that show a moderate resistance and/or bind to the antibiotic molecule and have the potential to evolve into resistance genes under selection pressures. Such genes are precursors to AR genes [128]. These AR genes and their precursors are located on the bacterial chromosome or may be encoded on resistance plasmids (R-plasmids), transposons, or integrons [130].

The environmental resistome is a source of mechanisms of antimicrobial resistance that can be acquired by commensal and pathogenic organisms [131]. AR genes transfer from donor to recipient bacteria of the same or different species via vertical transfer bacterial reproduction and horizontal gene transfer (HGT) via conjugation, transduction, and transformation [129,132,133]. Consequently, the resistome contributes to the development and progression of life-threatening diseases and renders them multi-drug resistant or extremely drug resistant [134].

Studies have demonstrated that various environmental niches have a distinct resistome that is influenced by anthropogenic activities to varying degrees [135,136,137,138]. For example, various AR genes were abundantly found in environments such as lakes [139], estuaries [140], rivers [136], and in waste waters originating from aquaculture [141], agriculture [142,143], and industrial sources [132,144,145]. The resistome of bodies of water with HABs has been studied [146].

As Gram-negative photosynthetic prokaryotes, cyanobacteria are more susceptible to selection pressures from antibiotics compared to other eukaryotic phytoplankton [147]. Cyanobacteria possess the mechanisms to contribute to the emergence and transmission of genes. They possess mobile genetic elements such as transposons, integrons, and the plasmids necessary for HGT [148]. Some cyanobacteria also demonstrate antimicrobial activity [149]. Hence, they inherently possess mechanisms for self-protection from these antimicrobials [150]. The evolution of these mechanisms can be a source of new AR genes in cyanobacteria. Further, higher concentrations of AR genes were found in cyanobacteria during algal blooms when compared to concentrations during non-algal-bloom periods [151]. The *Microcystis* and *Synechococcus* strains of cyanobacteria had significantly higher relative abundances of AR genes compared to other strains of cyanobacteria [151]. Thus, cyanobacteria are a reservoir of AR genes that may disseminate in the aquatic environment and further into other bacteria, especially gut bacteria and opportunistic pathogens such as *Vibrio vulnificus*, which are in close proximity to cyanobacteria [84,152,153]. Further, cyanobacterial toxins such as MCs cause gut dysbiosis. This opens a window for antibiotic-resistant opportunistic pathogens to colonize the gut and displace commensal bacteria in humans [111]. Alternatively, it can lead to the development of AR in commensal bacteria by the HGT of AR genes.

Mouse studies have shown that early life exposure to MC causes gut dysbiosis and increases the risk of acquiring AR [111]. The α-diversity of AR genes was significantly increased in fecal samples from mice exposed to MC compared to the control group. Tetracycline, aminoglycoside, macrolide, and glycopeptide are classes of antibiotics against which AR genes were abundantly found in groups exposed to MC. AR genes such as *mef*A and *msr*D, which confer resistance against macrolides by coding for efflux pumps, were significantly increased in the MC group compared to the control group. Another AR gene against macrolides, *mel*, also significantly increased with MC exposure. All these genes are known to be coded on the same mobile genetic element. Resistance against aminoglycosides and tetracyclines, conferred by *ant6* and *tet40*, respectively, increased in the MC group compared to the control. To identify the source bacteria causing the increase in AR, a gene provenance study was conducted. An increase in macrolide resistance via *mef*A, *msr*D, and *mel* was traced back to an increase in the bacteria *Bacteroides thetaiotamicron* in fecal samples from MC-exposed group. Similarly, an increase in aminoglycoside and tetracycline resistance via genes *ant*6 and *tet*O, respectively, was traced back to an increase in the bacteria *Lachnospiraceae bacterium* A4 in fecal samples from the MC-exposed group [111].

### 5.2. Vibriosis

*Vibrio* spp. is an estuarine marine bacterium ubiquitously present in all aquatic organisms and habitats [81]. Studies indicate a trend of an increase in the presence and rate of infection by *Vibrio* in the USA parallel to a trend in the increase of the atmospheric temperature [82]. Another factor that can affect the growth of *Vibrio* spp. Is salinity. With rising sea levels, the salinity of fresh and brackish waters systems is increasing [82]. This aids the expansion of *Vibrio* spp. into previously unreported aquatic systems such as coastal floodwaters, increasing the chances of human exposure via recreational activities and through drinking water. Therefore, climate change due to global warming is projected to greatly facilitate the growth of *Vibrio* spp. temporally and spatially [83].

There exist over one hundred *Vibrio* species, only a few of which are known to interact with humans. Some examples include *Vibrio cholerae*, *V. vulnificus*, V. *parahaemolyticus*, *V. alginolyticus*, *V. fluvialis*, and *V. anguillarum*. *V. vulnificus*, V. *parahaemolyticus*, and *V. alginolyticus* cause gastrointestinal disease, wound infections, and septicemia [154,155]. *V. cholerae* releases an exotoxin which leads to a gastrointestinal disease, “cholera”, that is characterized by watery stools [156]. *Vibrio vulnificus* is the cause of 95% of all seafood-related deaths in the United States [157]. Most infections by Vibrio spp. occur in months when waters are warm, such as from May to October [158]. *Vibrio vulnificus* and *Vibrio parahaemolyticus* have an optimum temperature for growth of ~37 °C [159,160,161]. Modes of entry for this bacterium include the consumption of contaminated seafood, wound infections, and primary septicemias from the ingestion of bacteria. Symptoms of a seafood-borne Vibrio infection include fever, chills, nausea, abdominal pain, hypotension, and the development of secondary lesions. Infections causing primary septicemia are most critical and have the highest mortality rate among other forms of infection [162,163]. Urgent antibiotic administration is critical for treatment, as a lack of treatment for more than 3 days is associated with an extremely high likelihood of mortality. Symptoms of wound infections by *Vibrio* spp. include fever, chills, edema, and cellulitis at the wound site that can progress to necrotizing fasciitis. Surgical procedures such as tissue debridement, skin grafts, and amputation are often required to manage the growing infection (58%). Mortality is relatively low in cases of wound infections (22%) [163,164,165]. Virulence factors responsible for the cellular damage and cytotoxicity exhibited by *Vibrio* spp. include hemolytic and proteolytic enzymes, such as hemolysin [166] and proteases necessary for tissue necrosis, cutaneous lesions, and increased vascular permeability causing edema [167,168]. *Vibrio* spp. also produce toxins, such as RtxA1, which are necessary for cellular lysis and tight junction disruption. This facilitates invasion of the bacterium into the bloodstream and across the intestinal barrier [169]. A loss-of-function mutation of the gene coding for RtxA1 demonstrated reduced liver damage upon infection by *Vibrio vulnificus* [170]. This highlights the toxin’s importance in the spread of the disease to the liver and its general role in the development of a systemic infection.

Patients with liver disease are more susceptible to a severe vibrio infection [171]. MC is a known hepatotoxin that aggravates liver disease [70,172]. This puts patients with underlying conditions such as alcoholic and non-alcoholic fatty liver disease at a higher risk of health effects from both Vibrio and MCs. Recent findings suggest that in mice, early life exposure to MC increases the risk of AR in gut bacteria, which persists in adulthood. The adult mice resistome showed an increase abundance of AR genes against antibiotics such as tetracyclines, aminoglycosides, macrolides, and glycopeptides [111]. Tetracycline is commonly used to treat Vibrio infections [111]. Thus, the introduction of tetracycline resistance in Vibrio via HGT from gut bacteria or from Cyanobacteria–Vibrio interactions in the common aquatic habitat could be a critical concern for the treatment of Vibrio infections as they can be fatal/ life-altering, especially in the case of primary septicemia and in wound infections.

### 5.3. Heat Stress

Another factor that contributes to a global climate change phenomenon are periodic heat waves. These heat waves can generate physiological heat stress, causing large-scale changes in ecological behavior and animal physiology. Heat stress can lead to abnormal pathology in humans, especially in children and the elderly. Interestingly, harmful algal blooms are prevalent in summer, and it is yet uncertain whether areas impacted by heat waves and with increased HABs may lead to an exacerbated health concern [173]. According to a large-scale, association-based study that encompassed 61.9% of an area representative of the population of the United States, the estimated fraction of deaths attributable to heat stress (HS) in the period of 1997 to 2006 was 0.44%. This corresponds to an average of approximately 5608 excess deaths every year due to heat in the period of 1997 to 2006 [174]. The Intergovernmental Panel on Climate Change’s Sixth Assessment Report (IPCC AR6) predicted an increase in the occurrences of heatwaves, prolonged warm seasons, and shorter winters. At a global warming rate of 2 °C, the heat wave extremes can have dire consequences for agriculture and health [175]. HS, or hyperthermia, triggers a thermal regulatory response in the human body, which includes cutaneous vasodilation and visceral vasoconstriction to aid heat dissipation [176]. HS also causes an acute phase response that involves the release of heat shock proteins (HSPs) such as HSP70, which have a protective role against apoptosis and necrosis due to HS [177].

HS has been shown to cause changes in the gut microbiota composition in rats [178], chicken [179], and pigs [180]. Gut dysbiosis due to a combination of HS and MC-LR may further increase the virulence of opportunistic pathogens, such as those belonging to *Vibrio* spp. Further, HS impairs gut integrity by increasing the permeability of the epithelial cells lining the gut lumen [181]. HS causes an increase in the uptake of intact proteins due to disrupted barrier function. MC exposure also causes the loss of gut barrier integrity [182]. This can further lead to the translocation of bacterial endotoxins and other inflammation-triggering moieties, causing increased systemic endotoxemia and complicating primary septicemia due to infection by *Vibrio* spp.

Although mild HS (37 °C to 38 °C) activates and enhances immune responses in the body, a rise in physiological temperatures beyond 38 °C is detrimental to the functioning of the immune system. Major HS (>38 °C) induces systemic inflammation and hindrances in the resolution of on-going inflammation. Physical labor or exercise under conditions of HS lead to higher levels of inflammation than those observed with exposure to HS only. Therefore, athletes, sportspeople, and laborers are more vulnerable to the ill effects of enhanced and continued inflammation in the body due to HS [173].

During HS, thermoregulatory processes cause the flow of blood from the viscera to peripheral circulation to aid in heat dissipation. This leads to hypoxic conditions in visceral organs such as the intestines, liver, and kidneys. Intestinal hypoxia can further induce local inflammation [183]. It would be interesting to consider a co-exposure model in which acute or sub chronic MC exposure during underlying heat stress can be studied. Though purely hypothetical at this point, coastal regions across the world are experiencing a slow rise in temperature and an increase in extreme heat days in combination with zones of hypoxia and pH changes in the water [184]. These regions have also seen HAB blooms and Vibrio cases [185]. Periodic heat waves, their effect in humans, and their health effects may exacerbate MC toxicity, although no detailed studies have been documented.

### 5.4. Cardiovascular Diseases

OATPs expressed in the heart and vascular system enable the cardio-toxic effects of MC-LR [186]. Acute intravenous exposure to purified MC-LR in male Fischer 344 rats caused a decrease in arterial blood pressure, heart rate, cardiac output, stroke volume, body temperature, oxygen consumption, and carbon dioxide production, indicating a reduced metabolic rate, in addition to an increase in arterial lactate concentrations [187]. Acute MC-LR exposure in female cross-bred pigs caused reduced mean aortic pressure, central venous pressure, and hepatic and renal hypoxia, causing circulatory shock [188]. Furthermore, MC-LR is known to cause structural damage to cardiomyocytes upon chronic exposure. In male Wistar rats, upon the intraperitoneal administration of MC-LR (10 µg/kg every 2 days for 8 months), micro-structural damage in heart tissue was observed, and there was loss of cellular cross-striations, an increase in mononuclear infiltrations in interstitial tissue, and fibrosis [122]. MC is known to cause oxidative stress in the heart through various mechanisms. There are reports that chronic and acute exposure to MC in animal models causes the destruction of cytoskeletal elements, thus damaging the physical structure of cardiomyocytes [189,190].

Another mechanism through which MC exposure can influence the outcome of cardiovascular disease (CVD) is through the unfolded protein response. The failure of the unfolded protein response proves to be a contributing factor to the development of CVD. The unfolded protein response is a defense mechanism by which cells prevent the accumulation of unfolded/misfolded proteins, thus preventing apoptosis. Under conditions of severe oxidative stress in the endoplasmic reticulum, the unfolded protein response may fail to prevent apoptosis, thus leading to damage to the cardiac tissue and cardiac failure [191]. MC is known to produce oxidative stress in the heart via various mechanisms [192].

MC can also induce cardiac toxicity through its primary target of inhibiting PP1 and PP2A. PP1 and PP2A regulate cardiac activities by dephosphorylating target proteins such as the voltage-dependent L-type Ca^2+^ (CaV1.2) channel and ATP-sensitive Na^+/^K^+^ channel. The inhibition of PP1 and PP2A by MC can disrupt the normal functioning of the heart [193,194].

MC can damage vascular tissue and cause hematological alteration. This damage to the vascular framework can affect the functioning of the heart, as indicated by decrease in the heart rate, cardiac output, oxygen consumption, and carbon dioxide production, indicating a reduced metabolic rate and progressive hypothermia [187]. MC-LR inhibited the proliferation, migration ability, and capillary-like structure formation of human umbilical vein endothelial cells in vitro. The structural and functional integrity of vascular endothelial cells is vital to the functioning of the vascular system [195,196].

### 5.5. Type II Diabetes

Type II Diabetes (T2D) is characterized by a progressive loss of insulin secretion by β cells in the pancreas due to insulin resistance (American Diabetes [197]). T2D is one of the largest epidemics in the world. Three major factors are responsible for the development and progression of T2D: genetic factors, lifestyle choices, and environmental factors such as food, toxins, and poisons [198]. An epidemiological study conducted near Lake Taihu, China, which is affected by frequent algal blooms, showed a higher incidence rate of T2D compared to other areas in China [199,200]. Exposure to water from algal blooms of the genus *Microcystis* in mice affected the expression of T2D-related genes and increased the incidence rate of T2D in mice [200]. Healthy mice were administered water from different regions of Lake Taihu, where algal blooms are a common occurrence. The mice were also acutely or chronically exposed to MC-LR, either orally or via intraperitoneal injection. Results showed that both mice, those administered water from algal blooms and those directly exposed to MC-LR, showed alterations in their glucose and lipid profiles. Both chronic and acute exposure to MC-LR caused a disruption of the insulin signaling pathway, which led to the upregulation of T2D-related genes, hyperinsulinemia, and insulin resistance. This toxic effect of MC-LR on glucose metabolism was brought about by targeting the hepatocellular mitochondria [200,201]. The effect of MC exposure on the insulin pathway is also mediated by inhibition of PP2A, as it is a critical regulatory phosphatase involved [202].

### 5.6. Non-Alcoholic Fatty Liver Disease

Non-alcoholic fatty liver disease (NAFLD) is a chronic liver disease. It is characterized by the deposition of fats in the liver that account for more than 5% of the liver weight and which cannot be explained by alcohol consumption [203,204]. In rare cases, the disease may progress from simple steatosis to hepatic fibrosis and liver cirrhosis. This progression is associated with increased malignant transformation in the liver, causing hepatocellular carcinoma [205] and/or liver failure [206]. A multi-hit hypothesis explains the progression of this disease to more severe forms [207,208]. MC-LR has been shown to be an enhancer of NAFLD progression in various pre-clinical models. MC-LR uptake proteins–OATPs—are abundantly found in the liver. In mice, MC-LR induced gut dysbiosis and gut barrier integrity impairment, which can cause the systemic circulation of inflammatory bacterial metabolites such as LPS. MC-LR-induced hepatitis -LR was marked by the activation of Kupffer cells, the resident liver macrophages, and the hepatic stellate cells primarily responsible for liver fibrosis. Kupffer cell activation further resulted in the release of pro-inflammatory cytokines such as MCP-1, IL-1β, and TNF-α [70].

In a preclinical investigation by Albadrani et al., it was discovered that MC targeted the liver via a NADPH Oxidase 2 (NOX2)–miR21 axis-dependent mechanism, which elevated oxidative stress, generated more peroxynitrite, and exacerbated NAFLD conditions [70]. The same team observed that early exposure to MC in juvenile mice led to insulin resistance, disrupted glucose transport, the early onset of liver disease. These effects were mediated by NACHT, LRR and PYD family pyrin domain-containing 3 (NLRP3)-inflammasome activation. Injury to liver parenchyma and mild fibrosis was observed in the livers of mice that were exposed to MC-LR early in life [125]. In mice, a high-fat diet typical to most Western countries exacerbated liver injury due to MC-LR, showed an increased expression of pro-inflammatory cytokines, and caused non-alcoholic-steatohepatitis-like symptoms, which is an advanced stage of NAFLD pathology [112,208]. In a study by Sarkar et al., wild-type male C57BL/6J mice exposed to MC (10 g/kg intraperitoneally, five doses per week for 2 weeks) had a modified gut microbiome with an increased abundance of Firmicutes and Proteobacteria and a concurrently decreased abundance of Bacteroidetes. In addition, it was found that mice with underlying NAFLD and who were treated with MC had a notably higher abundance of lactate-producing bacteria than the corresponding control group, which was associated with elevated NOX2 in the MC-exposed mice. The small intestine tight junction proteins were altered by MC exposure, resulting in a leaky gut; this, in turn, elevated serum endotoxemia, which later culminated in inflammasome-mediated intestinal inflammation, NOX2-mediated oxidative stress, and further gut dysbiosis [70,124].

**Figure 4:**
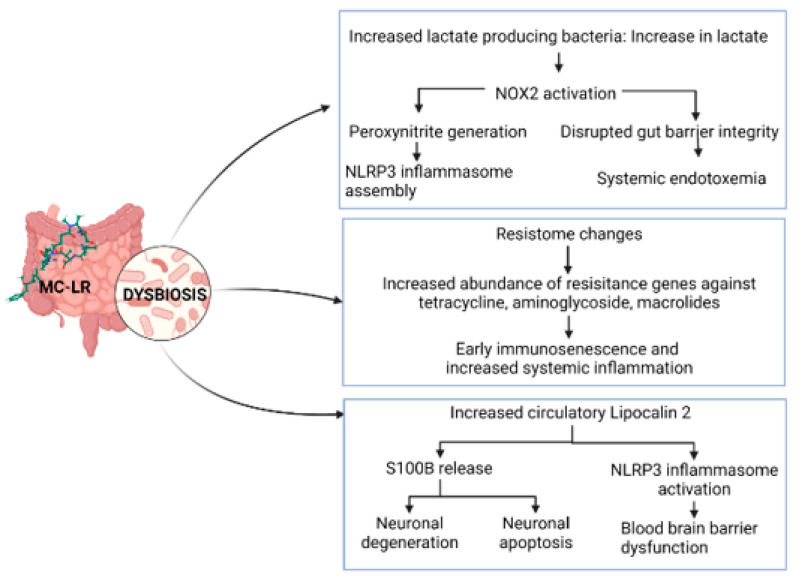
Microcystin-associated intestinal dysbiosis and its consequences.

Further, MC-exposed mice with underlying NAFLD showed signs of neuroinflammation, blood–brain barrier dysfunction, and neurodegeneration. MC exposure caused the decreased expression of blood–brain barrier tight junction proteins Claudin-5 and Occludin, which are essential for barrier integrity. Additionally, there was a marked increase in the expression of pro-inflammatory cytokines in the frontal cortex, causing an increased expression of lipocalin-2 (Lcn-2) and High mobility group box 1 protein. Circulating levels of S100B were high, indicating astrocyte activation. A mouse neuroblastoma cell line (Neuro-2a) exposed to mouse recombinant S100B or grown in a medium conditioned with a supernatant from primary mouse astrocytes exposed to MC or Lcn-2 showed a significant increase in cleaved caspase-3 and 3-nitrotyrosine levels. Thus, it can be concluded that MC-LR exposure with underlying NAFLD led to neuronal apoptosis via the release of S100B by astrocytes (Figure 4) [113].

## 6. Future Perspectives

As the rise of harmful cyanobacterial algal blooms is almost inevitable due to climate change, there is a dire need for advances in cyanotoxin monitoring, detection, and degradation technologies. Real time data collection systems such as automatic, high-frequency monitoring (AHFM) [209] and satellite-based monitoring systems that rely on satellite imagery to determine bloom intensities can overcome the limitations of manual sampling in collecting HAB-related data to some extent. These technologies can monitor multiple variables of the water system to reliably detect changes in the water quality, physical environment, biochemical cycles in response to anthropogenic activities, and changes in environmental conditions [209,210,211]. Advanced, software-based sensors (soft-sensors) and inferential machine-learning-based sensors can predict and raise alerts to aid in manual sampling to verify the status of the bloom [212]. Once a HAB has been detected, to assess the severity of the bloom, it is essential to detect the presence and concentration of various cyanotoxins. The development and use of reagent-free and reusable biosensors, such as modified, electrochemical aptamer-based biosensors, will reduce the time and cost of cyanotoxin detection [213]. Further, the microbial degradation of MC should be studied to understand the fate of MC in the aquatic environment. Identifying the key taxa responsible for MC degradation will help develop MC-removal technologies that are also sustainable [214].

We also need improved pre-clinical models and well-designed cross-sectional, population-based epidemiological studies to establish a better understanding of the consequences of MC exposure via drinking water, food, and recreational sources in healthy individuals as well as in individuals with underlying disease conditions. MC also has the potential to worsen outcomes of other diseases such as cardiovascular diseases, heat stress, type II diabetes, and non-alcoholic fatty liver disease. Epidemiological studies and research in relevant animal models are necessary to define the mechanism of the effects MC has on the pathogenesis of the above-mentioned diseases. MC exposure complicates the already-existing problem of antibiotic resistance. This can have severe outcomes in patients infected with opportunistic pathogens such as *Vibrio vulnificus, Vibrio parahaemolyticus*, and *Vibrio cholerae*. These pathogens significant as they are found in proximity to HABs.

As previously mentioned, gut dysbiosis is a feature of MC toxicity. Hence, more research should be focused on the development of therapeutic interventions directed towards maintaining gut homeostasis, such as prebiotics, probiotics, and synbiotics. Three human probiotics, *Lactobacillus rhamnosus* strains GG and LC-705 and *Bifidobacterium lactis* strain Bb12, can bind to MC-LR and can be used to remove MC-LR from water [215]. *L. rhamnosus* GG can bind and remove MC-LR from water with an efficiency of 60.73%. These three probiotic strains were synergistically more efficient at MC-LR degradation (~80%) when compared to individual strains [216]. This opens an array of opportunities to explore strains of probiotics for defense against MC in the gut as well as its degradation in water bodies.

Finally, data from HAB- and MC-related research should inform policy decisions. Better monitoring systems and efficient public communication of the relevant risks of the increase in HABs will help prevent adverse public health outcomes, especially in vulnerable communities. In summary, the above review provides a perspective of the impending climate change health effects due to harmful algal blooms that can be further complicated by factors, such as a warming temperature, rising sea level, coastal flooding, increased vibrio blooms, and resistance to antibiotics, following microcystin exposure. Further research in the above areas may help achieve a better understanding of their complicated relationships with climate change and what long-term effect they may have on human health.

## Figures and Tables

**Table 1:** Concentration of MC-LR in different drinking water reservoirs.

Country	Water Body	MC-LR Concentration	Reference
Brazil	Cascata water reservoir	~3 µg/L	[55]
	Guarapiranga water reservoir	~1 µg/L	[55]
	Amazon River	~1.4 µg/L	[59]
Canada	Quebec water reservoir	2.47 µg/L	[63]
Nigeria	Kubanni	2.8 µg/L	[64]
	Bomo	1.8 µg/L	[64]
Eastern Cuba	24 water reservoir	3.06 µg/L (in 5 lakes that exceeded WHO limits)	[57]
Morocco	Mansour Eddahbi lake	64.4 µg/g dry biomass weight	[61]
	Almassira lake	9.9 µg/g dry biomass weight	[61]

**Table 2:** Organ-specific toxic mechanisms of Microcystin, established using various animal models.

Target Organ	Model	Exposure	Observed Changes	Reference
Liver	Various	Various	Hepatic inflammation; activation of Kupffer cells and hepatic stellate cells; centrilobular apoptosis and necrosis; cytoplasmic vacuolization; cytoskeletal reorganization; hepatocyte blebbing; hepatocarcinogenesis.	[115,116,117], https://doi.org/10.1293/tox.14.259 (accessed on 10 April 2023), [118]
Kidney	Adult male Wistar rats	Chronic, sub-lethal dose, administered intraperitoneally	Glomerular collapse with thickening of the basement membrane; renal tubules showed actin condensation, appeared dilated and filled with proteinaceous casts; increased apoptotic cells in renal cortex and medulla.	[119]
Brain	Adult male BALB/c mice	Chronic sub-lethal dose, administered orally via drinking water	Disruption of blood–brain barrier function; neuroinflammation; microglial and astrocyte activation.	[120]
Lung	Specific-pathogen-free female BALB/C mice	Chronic low dose, administered orally in drinking water	Alveolar collapse and thickening of alveolar septum; slight increase cellular apoptosis at a higher dose; loss of epithelial barrier function.	[121]
Heart	Adult Wistar rats	Chronic	Increased size of cardiomyocytes; reduced myofibril volume fraction; fibrosis; mononuclear infiltration in interstitial spaces.	[122]

## Data Availability

Not applicable.
